# Developing an intervention to facilitate family communication about inherited genetic conditions, and training genetic counsellors in its delivery

**DOI:** 10.1038/ejhg.2015.215

**Published:** 2015-10-07

**Authors:** The Socio-Psychological Research in Genomics (SPRinG) Collaboration:, Ivan Eisler, Matthew Ellison, Frances Flinter, Jo Grey, Suzanne Hutchison, Carole Jackson, Louise Longworth, Rhona MacLeod, Marion McAllister, Alison Metcalfe, Trevor Murrells, Christine Patch, Stuart Pritchard, Glenn Robert, Emma Rowland, Fiona Ulph

**Affiliations:** 1 South London and Maudsley NHS Foundation Trust, London, UK; 2 Huntington's Disease Youth Organisation, London, UK; 3 Guy's and St Thomas' NHS Foundation Trust, London, UK; 4 Association for Multiple Endocrine Neoplasia Disorders (AMEND), Tunbridge Wells, UK; 5 Kings College London, London, UK; 6 Brunel University London, Middlesex, UK; 7 Central Manchester University Hospitals NHS Foundation Trust, Manchester, UK; 8 Cardiff University, Cardiff, UK; 9 Genetic Alliance UK, London, UK; 10 University of Manchester, Manchester, UK

## Abstract

Many families experience difficulty in talking about an inherited genetic condition that affects one or more of them. There have now been a number of studies identifying the issues in detail, however few have developed interventions to assist families. The SPRinG collaborative have used the UK Medical Research Council's guidance on Developing and Evaluating Complex Interventions, to work with families and genetic counsellors (GCs) to co-design a psycho-educational intervention to facilitate family communication and promote better coping and adaptation to living with an inherited genetic condition for parents and their children (<18 years). The intervention is modelled on multi-family discussion groups (MFDGs) used in psychiatric settings. The MFDG was developed and tested over three phases. First focus groups with parents, young people, children and health professionals discussed whether MFDG was acceptable and proposed a suitable design. Using evidence and focus group data, the intervention and a training manual were developed and three GCs were trained in its delivery. Finally, a prototype MFDG was led by a family therapist and co-facilitated by the three GCs. Data analysis showed that families attending the focus groups and intervention thought MFDG highly beneficial, and the pilot sessions had a significant impact on their family' functioning. We also demonstrated that it is possible to train GCs to deliver the MFDG intervention. Further studies are now required to test the feasibility of undertaking a definitive randomised controlled trial to evaluate its effectiveness in improving family outcomes before implementing into genetic counselling practice.

## Introduction

Families affected by Inherited Genetic Conditions (IGCs) face challenges in living with the condition and its risks in the present as well as attempting to manage the risk implications for future generations through reproductive decision making.^1, 2, 3, 4^ One of the biggest issues for parents is deciding when and how to talk to their children about the genetic condition, in an age and developmentally appropriate manner while promoting psychological well-being.^3, 4, 5, 6, 7, 8^

Most health professionals advocate that parents should begin to talk to their children about the IGC as soon as possible, gradually providing more information suited to their children's age and development.^6^ This assists children's maintenance of trust in their parents and affords them the opportunity to cope with and adapt to knowing about the IGC as they grow up, rather than finding out about the IGC in a dramatic and shocking way, which happens in many families.^3, 9^ Delayed or non-disclosure of genetic risk information reduces family cohesion,^10, 11^ which may result in family conflicts^3, 4^ and/or poor emotional and psychological well-being in families.^3, 9, 12^

Many parents want more support from health professionals about managing the IGC within the family and advice about talking to their children;^6^ however few have appropriate opportunities. Genetic counsellors (GC) are uncertain about how involved they should be in helping families to communicate risk information.^13^ Current practice focuses predominantly on the support of the individual affected or at risk, often to the exclusion of the wider family unit.^6, 13, 14^

In response to these findings we aimed to design an intervention that will assist parents and children in talking about and coping with the IGC affecting their family. Following a literature review and a discussion with senior family therapists, GC leaders, patient group representatives and researchers, we agreed that a multi-family discussion group (MFDG) intervention might be the most suitable model. MFDG interventions provide a safe context in which families can learn from and support each other, reduce their sense of isolation and stigma, and help to improve communication within families as well as between families and clinical staff.^10^ MFDG interventions are also potentially more cost-effective^11, 15, 16, 17, 18^ than one-to-one family therapy.

In MFDG settings, facilitators help families to share their experiences of living with their condition and find new and more effective ways of managing it. They have been successfully applied to a variety of mental,^15, 18, 19, 20, 21^ chronic health,^22, 23, 24^ familial cancer,^17^ and behavioural problems.^25^ MFDGs are based on the premise that families affected by the condition are better suited to understand and make suggestions to other families about how to cope and adapt,^10^ and the MFDG provides an environment to facilitate this constructive process.

Our research questions were ‘Are Multi-Family Discussion Groups a suitable intervention for facilitating family communication about an IGC?' and ‘Is it possible to train GCs to deliver the intervention?' The three study aims were (i) To ascertain whether a MFDG was a suitable approach, and acceptable to both families (parents, children, and young people) affected by IGCs and to GCs. (ii) To work with families and GCs to co-design the intervention to meet the needs of those receiving and delivering it and (iii) To train GCs in the intervention's delivery.

## Materials and methods

**Figure 1:**
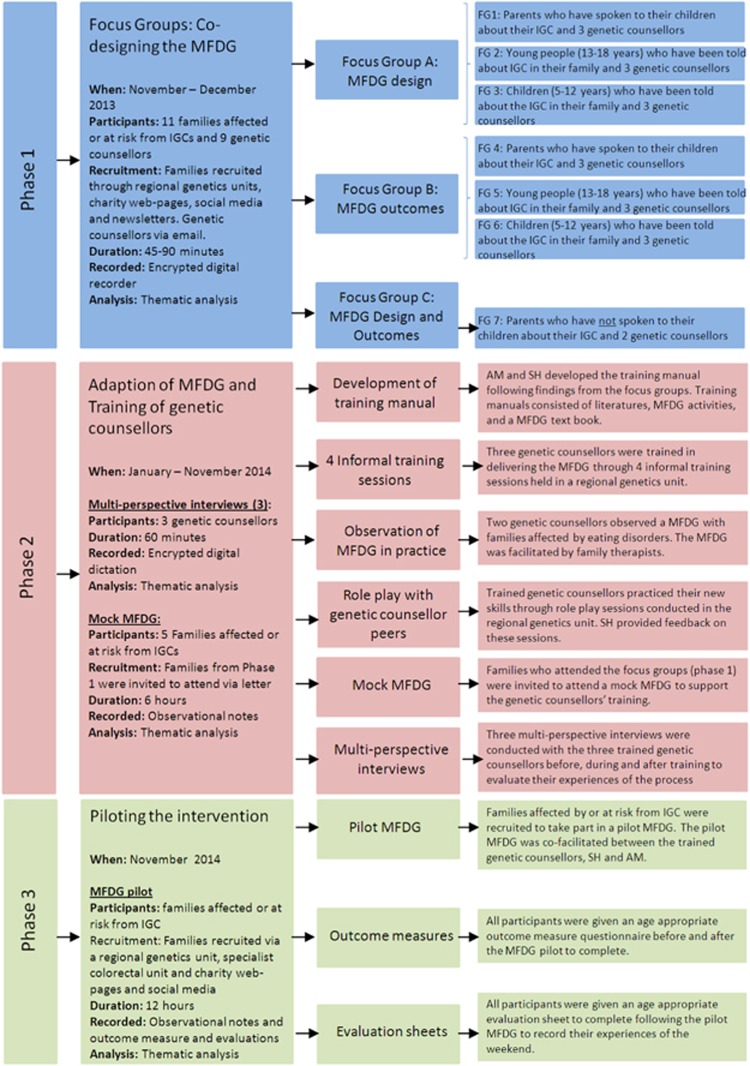
Diagram to show the three phases of the research project.

Following ethical approval by the NHS Riverside Ethics Committee, London; reference: 13/LO/0236, three methodological phases were undertaken to develop the intervention and pilot it with a group of families (see Figure 1).

### Phase 1—focus groups for co-designing the MFDG

Families, parents, children (5–12 years), and young people (13–18 years), affected by or at risk from an IGC were recruited via a regional genetics unit in the United Kingdom and advertisements on charity web-pages, social media, and newsletters. Interested families contacted the researchers and were provided with more study information. Potential participants were screened over the telephone to ascertain whether they had spoken to their children about the IGC in their family. If sufficient communication had taken place, that is, the genetic condition had been talked about with the children, then the family was invited to participate. All family members were sent information packs including recruitment letters, and consent/assent forms that were age and developmentally appropriate. Where communication had not taken place parents were invited to take part in an alternative focus group that did not include children or young people. GCs were recruited to the same focus groups by an email containing a recruitment pack.

Seven focus groups^26, 27, 28, 29^ were conducted with families affected or at risk from a range of IGCs and health professionals between November and December 2013 (see Figure 1). To stimulate ideas and discussions the research team presented the MFDG concept to participants^10, 15^ and asked families to comment and reflect on its potential use in the focus groups. Focus group A was divided into three concurrent focus groups: (i) parents, (ii) children, and (iii) young people with three GCs allocated to each group. The focus groups lasted between 45 and 90 min according to the participants' age/development stage. Child-centred methodologies were used with children and young people to support verbal communication.^30, 31, 32^ Families and GCs attending focus group A were invited back to participate in focus group B, to validate the findings from the original focus group (A) and to discuss the expected outcomes of the intervention and how these might be measured.

Families who had not yet spoken to their children and two GCs attended one focus group (C), which lasted 90 min and parents discussed both the design and the possible measurable outcomes of the intervention.

All focus groups were audio-recorded using encrypted digital Dictaphones and transcribed verbatim. Observational notes taken contemporaneously by the focus group observer and reflections made by the group leaders and facilitators were typed. Data were analysed using thematic analysis.^33^ Transcripts were imported into ATLAS Ti 6.2.28 for data management.^34^ Transcripts were read and re-read allowing the researchers (ER, CJ, and AM) to become familiar with the data and preliminary codes were noted.^35^ Codes were developed both inductively as they emerged from the data and deductively,^35^ drawing on knowledge and experience from previous research. Initial codes were discussed by two researchers (ER and CJ) and a code list and a code book were produced to create consistency in the coding process.^36^ Transcripts were coded by one researcher (ER), re-coded by a second (CJ), and reviewed by a third (AM). This data analysis procedure was followed for all interviews and focus groups in the three phases of work.

### Phase 2: Adaptation of the MFDG intervention and training of GCs

Two family therapists (IE and SH) and the research team used focus group findings (phase 1) to inform the adaptation of an MFDG for use with families affected or at risk of IGCs. With the intervention adapted, one family therapist (SH) and the study PI (AM) developed a training manual, which included family therapy concepts, techniques, explanations, examples of MFDG, and references to key texts. The training manual informed and guided the training of three GCs in MFDG therapy over a 6-month period.

The MFDG focused on four areas, which we had previously identified as causing families the most difficulty in talking about and coping with the IGC^3^ and which re-emerged in the above focus groups. The four areas foci were (i) setting the context and exploring families' experiences and knowledge of the IGC; (ii) the family's relationship with the IGC and how it affects their relationships with each other; (iii) coping with the emotions caused by the IGC and understanding each other; and (iv) recognising family members' strengths, building self-esteem, and focusing on how lessons learned can be used in the future. Group exercises and activities aimed at developing knowledge, skills, and confidence in relation to the four overarching areas were used with different stratifications of family members, parents, and children in their own peer groups, the family group or the whole group.

GC training involved four formal training sessions, role play, use of audio-visual materials and discussions. Training sessions were supported by the observation of MFDG's conducted with families affected by eating disorders, role plays with GC peers and the delivery of a small scale, 1 day mock MFDG conducted with families who attended phase 1 focus groups. The GC's experiences of training were captured via three multi-perspective interviews^37^ that were conducted by GR before, during, and after the training had taken place. Interviews were transcribed and imported into ATLAS Ti 6.2.28 for data management.^34, 38^ GC experiences of the MFDG training are reported elsewhere.^39^

### Phase 3—Piloting the intervention

Proceeding from the intervention's design and training of the GCs, families affected by or at risk from an IGC were recruited to attend a full MFDG programme, delivered by the trained GCs. Participants were recruited via a regional genetics unit, a specialist colorectal cancer unit, and advertisements on charity web-pages. Interested participants contacted the research team and were provided with verbal information and screened as in phase 1. Eligible families were sent information and consent/assent packs. Parents were given the option of inviting other individuals who were important in their family, for example, grandparents and parental siblings.

Activities and discussions were designed by SH and AM with guidance from IE to encourage family discussions around communication and functioning in relation to the IGC. The MFDG was observed by CJ who took contemporaneous field notes and AM recorded reflections in a research diary.

The purpose of the pilot MFDG was to ascertain whether the intervention was acceptable to families and if participants were willing and able to complete a battery of outcome measures, which will be used in a future randomised controlled trial (RCT). All participants were given an age appropriate booklet containing the validated family functioning and quality of life outcome measures which are being analysed and will be reported elsewhere once follow-up is complete. Qualitative outcome measures, feedback, evaluation data and observational notes of the research team were inputted into Atlas Ti 6.2.28 for data analysis.

## Results

We did not collect demographic information from participants but they varied in their socio-economic and educational backgrounds. Most participants but not all were white British or white Irish.

### Phase 1—Focus groups

Eleven families affected or at risk from IGCs and nine GCs participated in the focus groups (see Table 1). Seven families and nine GCs attended focus group A. Five participants, two parents, two children, and one young person, did not return to attend focus B due to ill health. Four families (five parents) and two GCs attended focus group C. Two main themes emerged that informed intervention design.

#### Theme 1: Clarifying the need for an intervention

Parents affected by or at risk from IGCs said that they faced challenges in disclosing genetic risk information to their children. Parents particularly perceived a deficiency of guidance and advice from health professionals in supporting them to talk to their children, which often led them to feel isolated and vulnerable:

*“There's no-one preparing us for [this], you've just got to muddle on...”* (Philip (Throughout this paper all names cited, except for the research team, are pseudonyms to protect the identity of participants.), Father of a Daughter affected by Vascular Ehlers Danlos Syndrome).

Parents were anxious that they did not have the right words, the knowledge or the appropriate skills to explain genetic risk information appropriately. Parents were worried about giving incorrect information or a false sense of hope. Furthermore, parents were concerned about their inability to rehearse or try out different ways of initiating conversations with their children.

“It's one of those things that you only get *one* chance [to do], you don't get to practice, unfortunately and it's that practice that you want... you can't practice on your five year old” (Jane, Mother, affected by Multiple Endocrine Neoplasia).

Children and young people reported feeling anxious, embarrassed or nervous in attempting to initiate conversations about how they felt. Simultaneously, parents felt that they did not have the emotional or psychological input to support them. Due to these challenges all participants (parents, children, and young people) reported that an intervention specifically designed for families affected by or at risk from IGCs was very necessary to facilitate family communication.

#### Theme 2: Designing the intervention

Parents, children, young people, and GCs were asked to co-design an MFDG intervention that would be acceptable to families affected by or at risk from IGCs and feasible to implement in NHS practice. Participants' discussions centred around two sub-themes; intervention logistics and the inclusion and exclusion criteria for attending families.

Intervention logistics. All participants saw value in the MFDG concept presented and were keen for the intervention to be more than a discussion group. Participants appreciated that engaging in activities would encourage discussions and help family members' understand each other's perspectives and feelings aroused by the impact of their IGC on family life. They also recognised the importance of engaging in activities to facilitate bonding with their own family members and also with the other MFDG families. Children and young people were keen to engage with dramaturgical or arts and crafts activities to keep them engaged and enthused but also to help them communicate their ideas and feelings during challenging discussions. The majority of parents saw the benefits to participating in activities for their children, but some parents thought that such participation could be embarrassing or uncomfortable for adults.

*John: “I think doing exercises...would be good for the children maybe...but I think that it would put some adults off”*

*Sarah: “Yes, we have enough of those team building meetings at work...I don't want this to be another”* (John, father of boy affected by Prader Willi Syndrome; Sarah, Carer for family members affected by and at risk of Huntington's Disease).

Participants discussed possible venues to host the intervention. All participants (parents, young people, and children) were adamant that hospitals or medical facilities should not be used because they conjure up memories. Participants preferred a neutral building that was welcoming, comfortable, and quiet. Young people wanted a venue that contained flexible spaces to move about in as it would prevent them from feeling pressured into participating in activities. Young people also expressed the need for break-out areas where they could talk to health professionals, other parents, or peers in private.

*“[It may] be helpful to have breakout areas...so if you think...I don't want to talk about these topics, but actually I've got this burning question you can go to another area”* (Jessica, young person at risk of Huntington's Disease).

Break-out areas were regarded as important by spouses and carers who talked about wanting to be able to engage in group discussions with peers who shared similar experiences, in the absence of their affected or at risk partner, so that they did not have to worry about saying something that would offend or upset them. Parents also saw value in their children talking and sharing experiences with their peers.

“*I can see a huge value with my child... being able to have a conversation with her age group... I think peer aged appropriate conversations would be very powerful because it is difficult for us, I mean who knows how to talk to an eight year old? Eight-year olds like talking to other eight-year olds*” (Jacob, Father of children at risk from Polycystic Kidney Disease).

Appropriate timing of the intervention was thought to be at weekends because evenings were tricky due to extra-curricular activities. Also families did not want to talk to their employers or their children's school to request time out to attend the MFDG due to fear of stigmatisation. Saturday afternoons with lunch were therefore preferred. The MFDG should be no longer than half a day in length and over several sessions, with parents, young people, and children agreeing that they may become tired, both physically and emotionally by the discussions and activities, causing difficulties concentrating if the MFDG was longer than half a day.

*“The idea of having a day which was 4–6 h long is way too long in my opinion, you'll have children getting exceptionally bored”* (Jack, young person at risk from Huntington's Disease).

Considering the frequency of sessions, participants felt that because discussions could be emotionally intensive, they did not want the sessions to be too close together. They wanted time to think, reflect, and perhaps implement some of the techniques. Conversely, they did not want the sessions to be too far apart because they would be unable to establish a rapport or friendships with the other families or lose interest or momentum in attending. Participants however were unable to come to an agreement about how many, the frequency and duration of the MFDG sessions. Parents did agree that intermediate support from their peer families would be valuable in implementing what had been discussed and shared in the MFDG rather than waiting for the next session.

*Inclusion and exclusion criteria for attending families:* Participants discussed which families and/or family members would be eligible to attend the intervention. Discussions centred on families' IGC, how much communication had already taken place within families and how soon after diagnosis families should attend the MFDG.

In exploring what IGCs should be included, participants, especially parents, initially felt that the MFDG should comprise families affected by or at risk from the same IGC. Participants initially advocated homogeneous MFDGs because they wanted to be able to share experiences with families whom they perceived to be in a similar situation and gain in-depth information about genetic inheritance patterns and risks. Furthermore, parents felt that an MFDG comprising different IGCs would confuse and upset their children.

“*I think...it's quite difficult...when people [don't] understand what other people [have got]...it's really hard to relate to what [their] problems and needs [are] compared to what...your family needs*” (Sarah, Carer for family members affected by and at risk of Huntington's Disease).

However, as discussions ensued, families' opinions about group composition evolved and participants thought that heterogeneous MFDG's would help them to learn about different viewpoints and experiences, which might be even more beneficial. They began to see communication challenges as similar regardless of the IGC and concluded that sometimes the differences could actually provide new perspectives and they would not be so focused on the disease but on the effects on the family.

“*I don't think it matters. I think the issue is that you've got a genetic disease that can be passed onto the children*” (Sarah, the above Carer in previous quotation).

Participants discussed how much communication was necessary before families could attend the MFDG. Parents who had not spoken to their children felt that no prior communication was required to be able to attend the MFDG as long as their children were absent. They believed there would be value in meeting other parents who had already initiated conversations, and felt that they could learn from that experience, helping them to gain confidence in initiating conversations with their own children. For the majority of participants however, they believed that a conversation needed to occur before attending the MFDG. The quality and quantity of this disclosure was however not specified with parents feeling that mixing parents at different stages of the disclosure process could be helpful in terms of receiving and sharing ideas. Some parents expressed concern that they often witnessed others who took polarised views in support groups about when and how children should be told about a specific IGC and this could cause friction. Parents from focus groups A, B, and C suggested those parents who had not talked to their children at all might need a different intervention.

Participants discussed what might be the appropriate time for families to attend the MFDG following diagnosis. The majority of parents agreed that the timing would depend on the individual's ability to cope with the information and whether they had already engaged in a conversation with their children.

*Philip...An experience on the day of diagnosis. It happened [around mid-afternoon] and by [early evening] we were driving around immediate family members... Some family members instantly filled up, ‘Right, what is it? How can we help?'...My brother, didn't show any emotion...It was six months later, [when] it hit him...*

*Alison: In some ways there wouldn't be any rules about whether it was immediate after diagnosis, it could be ten years or fifteen years down the line. There wouldn't be a rule about when you could access it... people come to terms with it at different points in time...*

*Jane: hmmm, I don't believe it really hit me until...about six weeks ago...I'm a very up, positive, happy person and I was just flat...and that was five years post-diagnosis...*

(Philip, Father of a Daughter affected by Vascular Ehlers Danlos Syndrome, focus group facilitator (AM) and Jane, Mother affected by Multiple Endocrine Neoplasia).

Finally, family members wanted to decide who attended the MFDG and whether they should invite extended family members or friends. Children and young people specifically wanted their friends to attend because they would be more comfortable talking to their friends than to their parents or siblings. Despite this, the majority of children and young people stated that being in an MFDG with their siblings was not a concern with the exception of one young person who suggested that he would be more comfortable and relaxed in talking to peers if his younger sibling was not present.

*“I guess it would come down to age, for example Phillipa and I, Phillipa would want me there if there were new people, more than I would want her there with me because I am a lot older than her... I maybe more comfortable talking without her”* (Jack, young person at risk of Huntington's Disease).

**Figure 2:**
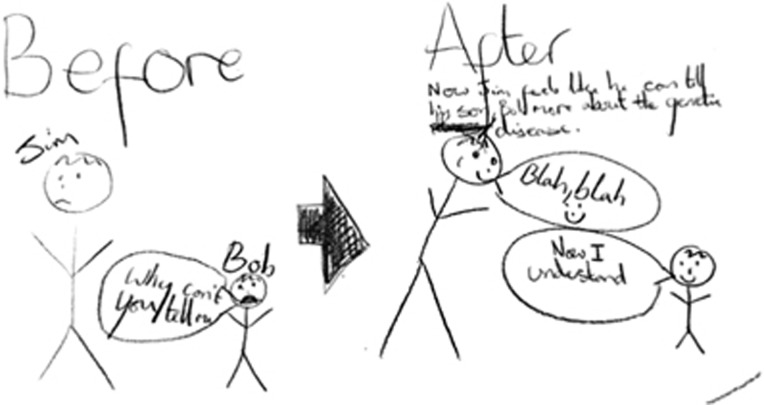
A picture showing a family before and after attending the MFDG intervention. In the before image the child is sad and confused asking their parent “why can't you tell me?” In the after picture the child is happier because he understands what is happening in his family because his parents have been ‘able to tell his son a bit more about the genetic disease'.

Concluding the focus groups, participants discussed the perceived benefits for families from attending MFDGs. For many participants gaining confidence and engaging in open communication with family members was regarded as the main benefits, by giving them the tools and methods to communicate more effectively with each other and build their confidence in posing and/or answering questions effectively (see Figure 2).

**Figure 3:**
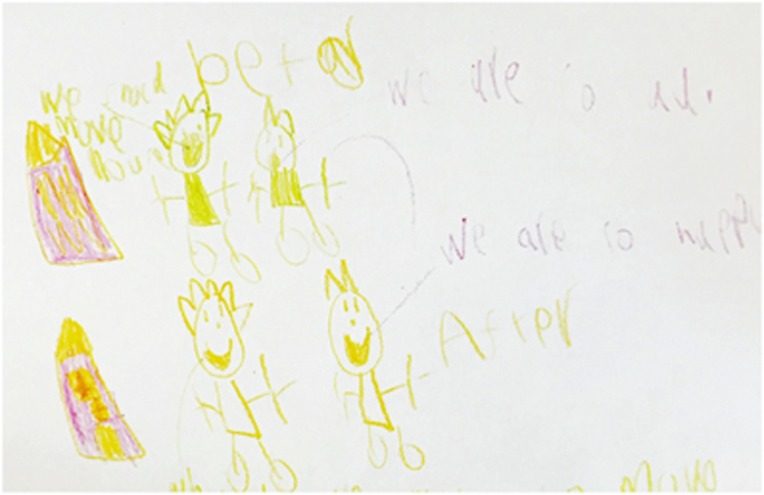
A picture showing the child's family before and after the intervention. In the before picture, the child has written by an image of his home ‘we are so sad' and in the after picture the child has written ‘we are so happy'.

Participants also perceived that MFDG attendance would lead to a happier home life with fewer arguments, more conversations and more family outings (Figure 3). Benefits to the health-care system were also acknowledged, with one young person stating that if the intervention was successful, it would reduce service use in other areas and therefore be cost effective for the health service.

*“You've also to be thinking from a health care point of view, if you get something like this right, you could be saving money and time further down the line and I think that's what the bigwigs need to understand. If you get something that saves the emotional side, half the time you'll save money later...it causes fewer problems if you just help someone talk”* (Jack, young person at risk from Huntington's Disease).

Logistical challenges such as travel costs and/or child care costs were also seen as a barrier for some families' participation. The most prevalent barrier to attending the MFDG however were parents' anxieties surrounding children's exposure to other IGCs. Parents perceived that being exposed to families with children affected by life-limiting conditions would upset their children.

*“If I went to the first group and I found out that there were families there with a terminal illness, I would be less inclined to go to another one because I wouldn't want to expose [my child] to those situations”* (John, Father of a Son affected by Prader Willi Syndrome).

### Phase 2: Development of intervention and training

The research team and family therapists used the focus group findings to adapt MFDGs for use with families affected or at risk from IGCs and three GCs were trained to deliver the intervention. Three multi-perspective interviews conducted before, during, and after the training captured the GCs shifting perspectives about the MFDG and their experiences of the training.^39^

In the first interview, the GCs were enthused about being trained in delivering the MFDG. They perceived that the training would improve their genetic counselling skills enabling them to counsel multiple families together and build their confidence in supporting families to communicate the risks associated with the IGC. However, they were also anxious because the training was very different from their current genetic counselling training and therefore would lead to a new and unfamiliar way of practicing genetic counselling in the future.

*Glenn: Why did you volunteer?*

*Katie (GC1): I think it seemed like a new skill and... It hasn't been done before....*

*Anna (GC2): Similar for me, [the research] sounded quite exciting, something completely new.... I also have a lot of concern about families communicating genetic results... so I want to know how to do it better and I thought I could learn a lot from this study not only skill wise but in terms of very specific genetic problems....*

*Glenn: Have you taken part in any similar training previously?*

*Anna (GC2): Nothing like this...*

*Rita (GC3): No I mean... it's part of our training as genetic counsellors to have some level of training in terms of communicating with families but....*

*Katie (GC1): ...not with family groups...*

*Rita (GC3): ...and although our title is genetic counselling and we all have genetic counselling skills, a lot of what we do is completely different to therapeutic counselling....*

(Multi-perspective interview 1 with GCs)

Following the commencement of training, the GCs participated in a second multi-perspective interview. At this time, all GCs were apprehensive about implementing the MFDG into practice. They were concerned about their ability and competence to facilitate the discussion groups and queried the recruitment of appropriate families to the intervention. There were also practical issues surrounding when these MFDGs should be conducted to accommodate the needs of families and also to assimilate with their department's current practice and organisational culture, as well as within wider NHS' financial and human resources:

*Glenn: In terms of them embedding it in routine practice in the service what do you think might be some of the obstacles to that, if any?*

*Katie (GC1): The obvious one that springs to mind is our lack of experience in running something like this, I know we get training for it but we will be under supervision when we do all of this and then it finishes and then we're kind of on our own and... that's one of my fears...*

*Anna (GC2): I think if we were to run our own multi family groups... it's so hard to get families with children... These are children who are in school... I don't really understand how you get all these families and professionals all to be together at the same time...it seems like a huge challenge.*

*Rita (GC3): Yet another barrier to this, a negative is just literally the environment that we're working in now. I mean we're all absolutely stretched, we're being asked to see more and more patients in less and less time and although we're all.... highly motivated, even just finding the time to do this kind of training is difficult and so I think that poses a huge challenge in terms of future care.* (Multi-perspective interview 2 with GCs).

Due to the GC' anxieties, apprehensions, and concerns, training took longer than anticipated. However, it was important that the GC felt confident in their ability to facilitate the groups before moving to phase 3. Confidence in the MFDG and their facilitation skills were enhanced through the observation of MFDG's with families in a service treating eating disorders, conducting MFDG activities with peers and co-facilitating a mock MFDG with families who had participated in phase 1. The family therapist (SH) debriefed the GCs following these activities to support their education and learning.

### Phase 3: Piloting the intervention

With the GCs confident in their ability to co-facilitate a MFDG, the intervention was conducted over one weekend in November 2014 and was attended by six families affected by or at risk from a variety of IGCs. The families comprised six parents and one step-parent, three grandparents, five young people (13–18 years), and three children (7–11 years) (see Table 1). Families participated in 12 h of scheduled MFDG activities over 2 days. The pilot intervention provided understanding of how the intervention could be refined for a future randomised control trial (RCT), to test its acceptability to families and the feasibility of delivering a definitive RCT with families affected.

Initially participants, especially young people and children, found the idea of sharing personal thoughts, feelings, and experiences with other families daunting or intimidating. However, through their interactions with other families and sharing their experiences with peers, who were able to understand and relate to what it is like living with an IGC, they thought the MFDG was hugely beneficial. The intervention therefore was not only considered acceptable but invaluable to their emotional well-being and their family's functioning.

*“It was a very good experience as Laura [grand-daughter] and myself were able to talk to other people in the group about our condition and they did not judge us and no blame was attached... To actually talk to people who knew what it was like to have genetic problems... we knew that we were not alone. So many people who do not have a genetic condition just do not understand how it feels and I have been told many times to “just get on with it” and “it's just life”, they have no idea what it is like or to lose a child through these conditions. The group discussions [have] been very welcome... in my life as I was able to talk about things I have not disclosed to anyone else. It felt a lot had been taken off my shoulders and I can go forward with a much positive view on life. This is a brilliant way of helping people with genetic conditions and I would definitely attend further sessions if these were available. I would highly recommend genetic group discussions for families with these conditions.”*

(Hollie, Grand-mother affected by Familial Adenomatous Polyposis).

Participants also recognised the benefit of the MFDG activities and exercises in facilitating communication and enabling them to understand issues and concerns from each other's perspectives as well as gaining advice from other families in how to manage challenges faced:

*“[The exercises] helped to bring the family together. They helped me to understand what [my] children are going through from their point of view and how to solve the daily hurdles*” (Jessica, Mother of a son affected by Familial Adenomatous Polyposis).

Having families with different IGCs was thought very beneficial by the participants because they heard and saw different ways of coping and talking about it. During the sessions parents reported how surprised they were that most of the issues they shared were similar, and usually focused on finding different ways of coping with the socio-psychological effects of the IGC on the family, rather than the IGC itself.

Participants recognised that the facilitator's role was important to the execution of the MFDG and to their outcomes. The majority of participants attending the pilot MFDG reported that the sessions had been facilitated well by the family therapist, research team and three GCs, stating that all the facilitators had been approachable and had the ability to manage group dynamics. This allowed participants to feel comfortable in sharing information with other families and management of potentially distressing situations. Participants thought it was important that the facilitators had both a good understanding of IGCs and an understanding of the wider social and emotional context.

Families' reported that they would recommend the intervention to others and would also want to attend future discussion groups as a ‘*graduate family'* to encourage other families to participate.

Following the MFDG, all three GCs reported that they were excited, motivated, and encouraged by their positive experiences of co-facilitating the MFDG and in witnessing the benefit to families. In the final multi-perspective interview, the GCs expressed their fervour for the MFDG and their eagerness to be involved in future research to facilitate the implementation of the MFDG into practice. They were also reassured that GC could be trained to deliver the intervention to families affected or at risk from IGCs.

*Katie (GC 1): I think we all weren't sure that it was really going to work, whether it would be beneficial to the families. I think by the end of [the MFDG] we all agreed that they did get something out of it and we got something out of it as well and it felt a lot more smoother, for whatever reason, and it just seemed like it flowed better and we were much better and more confident in what we were doing.* (Multi-perspective interview 3 with GCs).

## Discussion

The impact of genetic risk information on individuals and their families should not be underestimated, and finding ways of helping people to manage that information and to prevent it from disrupting their family functioning is essential as genetic testing becomes more routine. Simply giving people information is not sufficient, especially when many cannot take actions to alter the outcomes of the disease. It is imperative therefore to help families integrate the IGC into their lives while maintaining a cohesive and resilient family network to ensure they maintain a good quality of life, where the family's identity is not eroded or eclipsed by the IGC, as can so often happen.

Our previous work and that of others^3, 5, 6, 7, 9, 14, 40^ has shown the necessity of supporting families', to assist their adaptation to and coping with the IGC. This present research has now significantly progressed towards meeting this need by co-designing an intervention to promote and support family communication through adapting MFDGs co-facilitated by family therapists and GCs. With training, GCs were able to deliver such an intervention and deal with the complexity of socio-psychological issues that emerged during the sessions, demonstrating the acceptability of the MFDG to these health professionals. The families receiving the intervention also found it very beneficial and most have offered to act as future ambassadors and advocates to other families for future programmes, clearly demonstrating acceptability to families.

MFDGs need to be delivered at weekends as families do not want to talk to their employers or children's schools because they fear being stigmatised, an issue that was raised repeatedly during our MFDGs. Families also wanted to avoid hospital settings and these factors should be built into the design of future studies to test the effectiveness and economic viability of delivering the MFDG as part of future service provision. While there might be increased costs of delivering the intervention at the outset, in the longer term there are likely to be better outcomes for families and their individual members. Improved social-psychological care will lead to significantly reduced use of mental health and primary care services, which many families currently report they use as they struggle to cope.^3, 9, 12^

There were limitations to our work. The GCs required more training and development than we had originally anticipated, which we hypothesise is largely due to the limited training in family systems therapy and the lack of experience in working with multiple families in a single session. Further work is required to determine the future role of GCs in supporting family communication about IGCs because there is a risk in the future, with the advances on genomic medicine, for example, the 100 000 genome project in the United Kingdom, there may become a greater emphasises on genetic testing and this has to be done in parallel with attention on facilitating families' coping. The MFDG provides a comprehensive way of doing this. Further many families taking part reported never having the opportunity to receive genetic counselling and therefore offering these types of multi-family intervention may have an important role in facilitating these families' understanding and coping with the IGC. The research may also be limited by the sample of families participating, with families unwilling to communicate genetic information, unlikely to participate in the research.

Parents in the focus groups had difficulty in differentiating between the focus group they were in to design the intervention and the actual intervention. Most parents and young people reported therapeutic effects of simply taking part in the focus groups, despite the non-therapeutic role of focus groups being emphasised at the meetings. Parents' views on how the MFDG should be delivered, that is, a series of half day groups on a Saturday with lunch turned out to be inappropriate when it came to recruitment for piloting the intervention. Instead parents preferred one intensive weekend. On reflection, focus group parents were mirroring the focus group design and this requires consideration in designing future interventions because these participants could not envisage the MFDG, and following the half day mock session, all the participants wanted the day to go on longer. Therefore, some elements of co-design emerged beyond the focus groups.

## Conclusion

We have worked with parents, children, young people, and health professionals to co-design an MFDG intervention to facilitate better communication about an IGC in families affected by or at risk from an IGC, and demonstrated this to be acceptable and feasible. This is the first intervention of its kind for families affected by IGC. We are now working on the further evaluation and testing of the effectiveness and economic viability of the intervention before it is integrated into genetic counselling practice.

## Figures and Tables

**Table 1:** Participants per research phase

	*Research activity*	*Family/GCs*	*Inherited genetic condition*	*Participants*
Phase 1	Focus group A	1	Huntington's disease (HD)	Mother unaffected, son (aged 17) unaffected
		2	Polycystic kidney disease (PKD)	Mother affected, son (aged 11) affected by PKD
		3	Prader Willi syndrome (PWS)	Father unaffected by PWS, daughter (aged 9) unaffected by PWS
		4	Huntington's disease (HD)	Mother unaffected by HD, father affected by HD, son (aged 14) at risk of HD, son (aged 11) at risk of HD
		5	Huntington's disease (HD)	Mother unaffected, Son (aged 18) at risk of HD, Daughter (aged 12) at risk of HD
		6	Huntington's disease (HD)	Aunt/Carer, unaffected by HD, son (aged 18) at risk of HD, daughter (aged 11) at risk of HD
		7	Polycystic kidney disease (PKD)	Father, affected by PKD, mother, not affected by PKD, son (aged 7), at risk of PKD
		GCs	N/A	7 genetic counsellors from genetics departments across the United Kingdom
	Focus group B	2	Polycystic kidney disease (PKD))	Mother affected, son (aged 11) affected by PKD
		4	Huntington's disease (HD)	Mother unaffected by HD, father affected by HD, son (aged 14) at risk of HD, son (aged 11) at risk of HD
		5	Huntington's disease (HD)	Mother unaffected, son (aged 18) at risk of HD, daughter (aged 12) at risk of HD
		6	Huntington's disease (HD)	Aunt/Carer, unaffected by HD, son (aged 18) at risk of HD, daughter (aged 11) at risk of HD
		7	Polycystic kidney disease (PKD)	Father, affected by PKD, mother, not affected by PKD, son (aged 7), at risk of PKD
		GCs	N/A	7 genetic counsellors from genetics departments across the United Kingdom
	Focus group C	8	Polycystic kidney disease (PKD)	Mother, affected by PKD
		9	Multiple endocrine neoplasia (MEN)	Mother, affected by MEN
		10	Huntington's disease (HD)	Father, affected by HD
		11	Vascular Ehlers-Danlos syndrome (EDS)	Father, unaffected by V-EDS
		GCs	N/A	2 Genetic counsellors from London
Phase 2	Mock MFDG	2	Polycystic kidney disease (PKD)	Mother affected, son (aged 11) affected by PKD
		3	Prader Willi syndrome (PWS)	Father unaffected by PWS
		6	Huntington's disease (HD)	Aunt/Carer, unaffected by HD, son (aged 18) at risk of HD, Daughter (aged 11) at risk of HD
		7	Polycystic kidney disease (PKD)	Father, affected by PKD, mother, not affected by PKD, son (aged 7), at risk of PKD
		10	Huntington's disease (HD)	Father, affected by HD, grand-mother, not affected by HD
		11	Vascular Ehlers-Danlos syndrome (V-EDS)	Mother, unaffected by V-EDS, grandmother, unaffected by V-EDS
Phase 3	Pilot MFDG	3	Prader Willi syndrome (PWS)	Father unaffected by PWS, mother unaffected by PWS, Son (aged 10) affected by PWS, Daughter (aged 9) unaffected by PWS
		12	Ehlers-Danlos syndrome (EDS)	Mother affected by EDS, daughter (aged 11) at risk of EDS
		13	Von Hipple Lindau disease (VHL)	Mother affected by VHL, daughter, unaffected by VHL
		14	Familial adenomatous polyposis (FAP)	Grandmother, unaffected by FAP, granddaughter (aged 16) affected by FAP
		15	Familial adenomatous polyposis (FAP)	Mother, unaffected by FAP, stepfather, unaffected by FAP, son (aged 15), affected by FAP
		16	Ehlers-Danlos syndrome (EDS)	Mother, affected by EDS, daughter, at risk from EDS, daughter, at risk from EDS, grandmother, unaffected by EDS, grandfather, affected by EDS
